# Renal Lesions in Horses with Oleander (*Nerium oleander*) Poisoning

**DOI:** 10.3390/ani12111443

**Published:** 2022-06-03

**Authors:** Chelsea A. Sykes, Francisco A. Uzal, Aslı Mete, Jennine Ochoa, Michael Filigenzi, Robert H. Poppenga, Javier Asin

**Affiliations:** 1 California Animal Health and Food Safety Laboratory System (CAHFS), University of California-Davis, Davis, CA 95616, USA; casykes@ucdavis.edu (C.A.S.); fauzal@ucdavis.edu (F.A.U.); amete@ucdavis.edu (A.M.); jnochoa@ucdavis.edu (J.O.); msfiligenzi@ucdavis.edu (M.F.); rhpoppenga@ucdavis.edu (R.H.P.); 2 CAHFS Davis Branch, Davis, CA 95616, USA; 3 CAHFS San Bernardino Branch, San Bernardino, CA 92408, USA; 4 CAHFS Tulare Branch, Tulare, CA 93274, USA

**Keywords:** horse, oleander, kidney, renal lesions

## Abstract

**Simple Summary:**

Oleander (*Nerium oleander*) poisoning is diagnosed postmortem based on gross and microscopic changes in the heart combined with the identification of oleandrin in samples from the affected animals. Several studies in multiple mammalian species have identified microscopic lesions in the kidneys as well, although this has only been briefly mentioned in horses. In this study, we retrospectively reviewed several cases of spontaneous oleander poisoning in horses, described gross and microscopic lesions in the kidneys, and assessed the prevalence of such microscopic lesions compared with other causes of death in order to determine if they can be used as a diagnostic marker of oleander poisoning in horses. Microscopic kidney lesions were detected in horses with oleander poisoning and were similar to the changes documented in other mammalian species, although the frequency and severity were generally lower. Similar renal changes could be detected in horses that died spontaneously due to other causes or that were euthanized. We concluded that microscopic kidney lesions occur in horses with oleander poisoning, but these are not a specific diagnostic marker to differentiate it from other disease processes.

**Abstract:**

A presumptive postmortem diagnosis of oleander (*Nerium oleander*) poisoning is made based on the histological observation of cardiomyocyte degeneration and necrosis, which is considered to be a reliable diagnostic marker, and can be confirmed via the detection of oleandrin in tissues or fluids. However, cardiac lesions may not be present in every case, and autolysis can often preclude the identification of subtle changes in the cardiomyocytes. Several studies of experimental oleander poisoning have noted the presence of renal lesions in multiple mammalian species, and case studies of accidental exposure have found similar, although more variably severe, renal abnormalities. Kidney pathology in horses with oleander poisoning has been only briefly mentioned. In this study, we reviewed 21 cases of spontaneous oleander poisoning in horses, evaluated the kidneys microscopically, and compared the renal microscopic lesions with those detected in 10 horses that died or were euthanized due to other causes to assess if histological renal changes could serve as an additional diagnostic marker for oleander poisoning in horses. We found that microscopic renal lesions, principally mild to moderate tubular changes such as hyaline cast formation, neutrophilic casts, epithelial attenuation and necrosis, as well as mineralization and congestion, occur in horses with oleander poisoning. Most of these changes match the descriptions of lesions previously noted in other species, although with less frequency and severity. Similar lesions were found in horses that died spontaneously due to different causes or were euthanized. We concluded that microscopic renal lesions may be detected in horses with oleander poisoning but they cannot be used as a diagnostic marker that allows differentiation from other disease processes or causes of death.

## 1. Introduction

Oleander (*Nerium oleander*) is a hearty evergreen plant that originated in the Mediterranean region and has been long associated with intentional and unintentional poisoning of humans and animals [1]. Due to its drought-resistant nature, oleander has become a popular ornamental plant throughout the western and southern United States and elsewhere in the world [1,2]. As its range has expanded, so, too, have the reports of intoxication. All parts of the plant are poisonous, containing varying amounts of cardiac glycosides known as cardenolides, the most common of which is oleandrin [1,2]. Although the toxicity varies depending on the flower color and time of year, as little as 0.005% of the body weight (i.e., a handful of leaves) is typically sufficient to cause fatal intoxication for most mammals [1,2,3]. The mechanism of action of the cardenolides is inhibition of the Na^+^/K^+^-ATPase transporters, primarily in the heart, which hinders the cardiomyocytes’ ability to maintain the electrochemical gradients required for calcium movement and cell contraction. This results in a net calcium elevation inside the cardiomyocytes, which leads to cell death and secondary arrhythmias [1,4]. The exact toxicokinetics have not been fully elucidated, though it is known that the cardenolides are absorbed rapidly in the gastrointestinal tract and appear to be distributed widely throughout the body [2,5,6]. Most cardenolides, including oleandrin, are primarily excreted via the hepatic route, with some minor excretion occurring through the renal route [2,5,6]. Cardenolides have occasionally demonstrated primarily neurotoxic effects, as noted in chickens, and may affect Na^+^/K^+^-ATPase transporters in organs other than the heart in some species [1]. Digitalis, a cardenolide found in *Digitalis purpura*, is known to have a diuretic effect by direct action on the Na^+^/K^+^-ATPase transporters in renal tubules [7].

Oleander is bitter tasting, and most animals avoid consuming it unless no other forage options are available [1]. The most common route of exposure in livestock and horses is by accidental incorporation of oleander into hay bales or clippings that are being thrown into a pasture or pen [2,5,8,9]. As it dries, the plant becomes less bitter but remains toxic, and the animals are less likely to avoid the clippings [1,2]. The common clinical signs of oleander poisoning in horses include colic, weak pulse, congested mucous membranes, slow capillary refilling time, tachycardia, high-grade heart blockage, depression, lethargy, tremors, acute renal failure, and death [1,2,5]. It is not unusual for owners to find animals dead without prior clinical signs being observed.

Exposure to oleander is confirmed by finding leaves in the gastrointestinal tract and by testing serum, plasma, urine, liver, or gastrointestinal contents for oleandrin [2,5]. A definitive postmortem diagnosis of intoxication should consider the history of exposure, clinical signs, and microscopic lesions of cardiomyocyte degeneration and necrosis found in the heart, which are considered a useful diagnostic marker of oleander poisoning. However, in some cases, there may be minimal microscopic changes in the heart, and it has been theorized that acute ingestion of large doses can be fatal without causing significant microscopic structural alterations to the cardiomyocytes, which can hamper the diagnosis [1,2,3,5]; in addition, identification of cardiac lesions can be challenging in carcasses with advanced postmortem decomposition.

The cardiac lesions due to direct damage of the cardenolides on the myocardium have been well described in several species, as well as the gastrointestinal lesions associated with the irritant effects of the plant on the mucosae, and the pulmonary lesions that result from heart failure secondary to the cardiac damage [2,3,5,8,10,11,12]. In goats, sheep, and cattle, renal lesions have also been well described, and appear to occur commonly and with moderate to marked severity in experimental oleander intoxication [8,11,12,13]. Mild, diffuse renal lesions were found in all necropsied cattle in a study of accidental exposure by Ceci et al. [8]. More variable renal lesions were noted in three necropsied New World camelids in a different study of accidental exposure [9]. In a retrospective study that included 30 cases of natural oleander poisoning in horses from California, the renal lesions were just briefly mentioned and were noted to have occurred in the majority of necropsied cases and grouped together under the same category that included necrosis, infarction, tubular mineralization, and congestion [10].

Direct damage to the kidney has been documented for one cardenolide [7], so it is possible that other cardenolides such as oleandrin are able to directly damage renal cells. Alternatively, as oleander poisoning results in cardiac dysfunction with systemic circulatory compromise, some of the kidney lesions could be secondary to renal hypoperfusion, since the cortex is very sensitive to the effects of ischemia [14]. Both mechanisms of renal impairment could occur simultaneously, and if the patterns of damage were consistent, these histological renal lesions could serve as another diagnostic marker of oleander poisoning.

To the authors’ knowledge, literature characterizing renal lesions in horses with oleander poisoning does not currently exist. This study aimed to (1) describe the type, extent, and frequency of gross and microscopic renal lesions in horses that died with a diagnosis of oleander poisoning, and (2) establish whether microscopic renal lesions can be used as a postmortem diagnostic marker of oleander poisoning in horses.

## 2. Materials and Methods

### 2.1. Case Selection

Necropsy cases that were submitted to the California Animal Health and Food Safety Laboratory system (CAHFS; University of California-Davis) between 2007 and 2021 were reviewed for inclusion in the study. The selection criteria included being a horse submitted for necropsy, testing positive for oleandrin, and having evaluable hematoxylin and eosin (HE)-stained kidney histological slides; animals with available slides but with advanced renal autolysis were excluded. In total, 21 cases were selected. The reports were reviewed, and the case signalments and the renal gross findings in each case were recorded. Non-renal gross and microscopic lesions related to oleander poisoning [10] were reviewed briefly for each case. The method and sample type used to confirm exposure to oleander was determined.

Ten controls were selected for a comparison of renal histology. The selection criteria for the control cases included being a horse submitted for necropsy to CAHFS and having evaluable HE-stained kidney histological slides, in addition to either being euthanized (five control cases) or dying with a disease process that involved systemic cardiovascular compromise (five control cases).

### 2.2. Oleander Testing Methods

Oleander exposure was confirmed by identifying oleandrin by extraction from samples of the serum, gastrointestinal contents (including the contents of the stomach, colon, or cecum), liver, or urine, and analysis by liquid chromatography-mass spectrometry (LC-MS/MS) following CAHFS’s standard operating procedures (SOPs) and using previously published methods [15]. Briefly, samples were solvent-extracted and concentrated prior to LC-MS/MS analysis. Tissue sample extracts were subjected to an additional cleanup procedure using Florisil solid phase extraction prior to the analysis.

Extracts were analyzed using a Sciex 4000 Qtrap system (Sciex Corp, Toronto, ON, Canada) equipped with a 150 × 4.6 mm, 5 µm Luna C18 (Phenomenex Corp, Torrance, CA, USA) column. High-pressure liquid chromatography (HPLC) mobile phases consisted of water and methanol, each with 0.1% formic acid, and gradient elution was utilized. Oleandrin was identified by matching the retention times and MS/MS spectra from analytical standards with those from the samples.

The reporting limits for oleandrin for each matrix are as follows: serum and urine, 5 ppm; chyme samples of gastrointestinal contents, 50 ppm; liver, 5 ppb.

### 2.3. Microscopic Renal Evaluation and Scoring

For each case, kidney histological slides were prepared as part of the diagnostic workup following CAHFS’s SOPs. Briefly, tissues were fixed in 10% neutral-buffered formalin for 24 h, paraffin-embedded, sectioned at 4 µm, and stained with HE.

For this study, a re-evaluation of the HE-stained kidney histological slides was performed blindly by one of the authors (JA). A standardized set of microscopic findings was established based on the renal structure affected as follows [8,10,11,12,13,14]: tubules: intratubular hyaline casts, intratubular neutrophilic casts, tubular epithelial attenuation, tubular epithelial necrosis, and tubulointerstitial mineralization; glomeruli: proteinaceous eosinophilic material in Bowman’s space; interstitium: lymphoplasmacytic infiltrates, hemorrhages; vasculature: congestion.

These changes were recorded and scored according to a grading scale based on severity (−: absence; +: mild; ++: moderate; +++: severe).

### 2.4. Statistics

The absolute and relative frequencies of the “absence” (−) or “presence” (+, ++, and +++ pooled) of each finding in the oleander-intoxicated and control groups were described using 2 × 2 contingency tables, and Fisher’s exact test was used to establish whether there were any statistical differences in the frequencies observed between both groups. Data were analyzed with IBM SPSS 19.0 for Windows (IBM Corp, Armonk, NY, USA). A *p*-value of ≤0.05 was considered statistically significant.

## 3. Results

### 3.1. Case Signalments, Lesions in Non-Renal Organs, and Oleandrin Detection

The details of the 21 cases of oleander poisoning (Cases 1 to 21) that fulfilled the inclusion criteria and of the controls (Controls 1 to 10) are included in Table 1. In 18/21 (85.7%) cases of oleander poisoning, a specific age was available and ranged from 3 months to 18 years (mean: 8.7 years). The exact age was not available for 3/21 (14.3%) animals; two of those animals were recorded as “adults”. Four of the 21 (19%) oleander-intoxicated horses were euthanized by barbiturate overdose and 17/21 (81%) died spontaneously. Of the 10 control cases, 5/10 (50%) were euthanized due to different disease processes (Controls 1–5) and 5/10 (50%) died spontaneously due to causes that involved acute or subacute systemic cardiovascular compromise (Controls 6–10).

**Figure 1:**
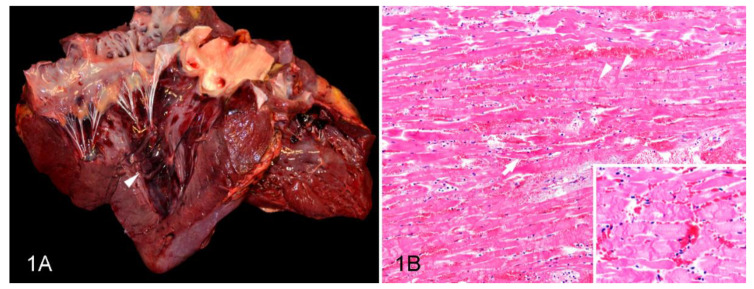
Cardiac lesions in a horse with oleander poisoning; case 20. (**A**) Gross aspect of the heart. Multiple hemorrhages on the endocardium and subendocardial myocardium. The left papillary muscle has been sectioned and the hemorrhages can be seen penetrating deep into the myocardium. (**B**) Microscopic changes in the myocardium. Cardiomyocyte degeneration and necrosis characterized by swelling, hypereosinophilia, loss of cross-striations with fragmentation (arrow), hypercontraction bands (arrowheads), and interstitial hemorrhage and edema. Inset: Detail of the hypercontraction bands. Hematoxylin and eosin.

All oleander-intoxicated horses had one or more of the following gross and/or microscopic non-renal lesions previously associated with oleander poisoning in horses [10]: myocardial and endocardial hemorrhages, cardiomyocyte degeneration and necrosis, edema, and/or neutrophilic infiltrates (Figure 1A,B); pulmonary edema, congestion, and/or hemorrhages; enterocolitis, watery intestinal contents, and/or submucosal edema; and congestion and hemorrhages in multiple organs. Out of these non-renal lesions, the most common and diagnostically relevant were the cardiac changes, which were detected in all but one (Case 21) of the cases reviewed.

Oleander exposure was confirmed by the detection of oleandrin in either the serum (1/21 (4.8%); Case 1), gastrointestinal contents (14/21 (66.7%); Cases 2–10, 13, 14, 16, 17, and 20), liver tissue (5/21 (23.8%); Cases 11, 15, 18, 19, and 21), or urine (1/21 (4.8%); Case 12). The samples of the gastrointestinal contents included the stomach contents (11/21 (52.4%); Cases 3, 4, 6–10, 13, 14, 17, and 20), cecum contents (2/21 (9.5%); Cases 5 and 16), and colon contents (1/21 (4.8%); Case 2).

### 3.2. Renal Lesions

**Figure 2:**
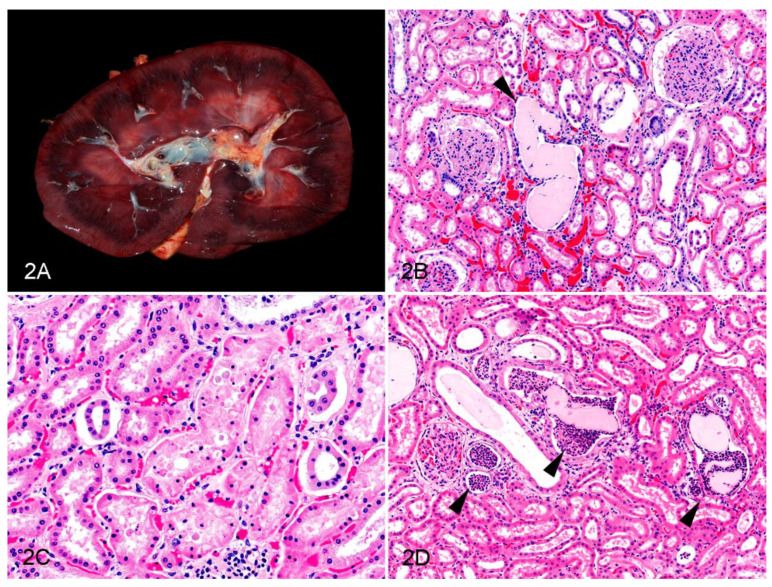
Renal lesions in horses with oleander poisoning. (**A**) Gross aspect of the kidney, showing marked corticomedullary congestion; Case 20. (**B**–**D**) Microscopic changes in the renal cortex (Hematoxylin and eosin). (**B**) Dilated tubule with a hyaline cast and attenuated epithelium (arrowhead); Case 15. (**C**) Tubular epithelial necrosis with hypereosinophilia, cellular fragmentation, and pyknosis; Case 20. (**D**) Neutrophilic casts within the tubular lumina (arrowheads). Some hyaline casts are mixed with the neutrophilic aggregates; Case 8.

The necropsy reports described gross kidney lesions in 7/21 (33.3%) cases of oleander poisoning. These included congestion (2/21 (9.5%); Cases 3 and 20; Figure 2A), white streaks in the cortex (1/21 (4.8%); Case 18), pale kidneys (3/21 (14.3%); Cases 7, 12, and 15), swollen kidneys (1/21 (4.8%); Case 8), misshapen kidneys (1/21 (4.8%); Case 7), and hemorrhage in the outer medulla (1/21 (4.8%); Case 3).

The results of the microscopic examination are presented in Table 2. Intratubular hyaline casts were the most common finding in the tubules, as these were present in 17/21 (81%) of the oleander intoxication cases; this change was present both in the cortex (in both the proximal and distal tubules) and in the medulla (Figure 2B). Tubulointerstitial mineralization was detected in 13/21 (61.9%) cases, principally in the corticomedullary junction and in the medulla. Tubular epithelial attenuation and tubular epithelial necrosis were less commonly detected (in 9/21 (42.3%) and 5/21 (23.8%) cases, respectively; Figure 2B,C), and were observed in the cortex (the former in both the proximal and distal tubules, and the latter mostly in the proximal tubules) in most of the cases. Intraluminal tubular neutrophilic casts were found in the cortex, in both the proximal and distal tubules, in 4/21 (19%) cases (Figure 2D); in some of these cases, the neutrophils intermixed with hyaline casts and sloughed cells in the tubular lumina. Within the glomeruli, proteinaceous eosinophilic material in Bowman’s space was noted in 6/21 (28.6%) cases, whereas in the interstitium, lymphoplasmacytic infiltrates were noted in 5/21 (23.8%) cases and hemorrhages in 4/21 (19%) cases. Congestion was noted in all 21 (100%) cases. The severity of these changes ranged from mild to moderate in the vast majority of the cases, except for congestion, which was severe in 5/21 (23.8%) cases, as well as severe hyaline cast formation, tubulointerstitial mineralization, and interstitial hemorrhages, which were detected in 1/21 (4.8%) case each. Except for intraluminal neutrophilic casts in the tubules, which were not present in any of the control horses, similar microscopic changes as detected in the oleander poisoning cases were also seen in the kidneys of the control animals (Table 2). Oleander poisoning cases tended to have more intratubular hyaline casts (17/21 (81%) versus 6/10 (60%) in the controls) and tubulointerstitial mineralization (13/21 (61.9%) versus 4/10 (40%) in the controls). There were no statistically significant differences in the frequencies of any of the evaluated features between the oleander intoxication cases and the controls.

## 4. Discussion

Oleander poisoning has been associated with acute renal failure in several species. In horses, renal lesions have been only briefly mentioned [10]. This study aimed to describe gross and microscopic renal lesions in horses with accidental oleander intoxication. Heart lesions of cardiomyocyte degeneration and necrosis are useful diagnostic markers of oleander poisoning, although they may be subtle in some cases or even difficult to identify in animals with advanced autolysis. Therefore, this study also aimed to establish whether the identified renal changes, especially the microscopic findings, could be used as a reliable diagnostic marker of oleander poisoning that could presumptively differentiate it from other disease processes and justify further confirmatory toxicological testing.

All the horses with oleander poisoning included in this study had several gross and/or microscopic changes in the kidneys. Gross findings were rare and generally non-specific. Similar non-specific gross renal lesions were found in goats, sheep, and cattle intoxicated with oleander in three studies [8,11,12], but were absent in a different study on oleander-intoxicated goats by Barbosa et al. [13]. Other studies did not mention if renal gross lesions were present or absent in horses or livestock with oleander intoxication [3,10,16]. This lack of consistent and unique gross lesions suggests that the renal macroscopic findings cannot be used as a diagnostic marker for oleander poisoning.

Histological renal lesions were noted in every oleander-intoxicated horse included in this study. Congestion, intratubular hyaline casts, and tubulointerstitial mineralization were the most common microscopic changes noted. Several changes were found in the cortical tubules, including hyaline cast formation, tubular epithelial attenuation, neutrophilic casts, and tubular epithelial cell necrosis, all of which pointed to tubular damage. This is not unexpected, as the renal tubules, especially the proximal convoluted tubules, are the area of the nephron most susceptible to ischemia and to the action of toxicants [14]. Overall, these microscopic findings suggest a degree of tubular impairment, with epithelial degeneration, proteinosis, epithelial necrosis, and possible sloughing of cells to the lumina with eventual neutrophilic migration [17,18]. These abnormalities may explain some of the alterations in the biochemical renal parameters, including elevated serum creatinine and blood urea nitrogen, as reported by Renier et al. [10].

The renal microscopic lesions found in the equine cases of oleander poisoning match the findings described in sheep, cattle, and goats [8,11,12,13]. However, the lesions noted in these studies were consistently more severe than what we found in the equine cases. One possible explanation for this disparity is the ingested dose of oleandrin. The sheep and goats in the abovementioned studies were all experimentally intoxicated with a similar lethal dose of oleandrin, noted as being two times the reported lethal dose for ruminants [11,12]. Another experimental study in goats included multiple oleander doses at the lethal ruminant dose for 6 days, and the final dose was two times higher [13]. The horses in the cases reviewed here were all associated with accidental ingestion of oleander, and the ingested dose of oleandrin likely varied significantly between cases. It is also possible that more severe and varied renal changes would have developed in some of the euthanized animals if they had been allowed to die spontaneously.

Similar microscopic changes to those described in oleander-intoxicated horses were detected in the control cases of this study, including horses that died spontaneously and those that were euthanized. This is different from the previous reports on sheep and goats [11,12,13], which found that the controls showed few to no renal microscopic lesions, possibly because, in those studies, the controls were healthy experimental animals that were euthanized for the purpose of the trial. However, in our study, there were some trends, including a mild increase in the frequency of hyaline casts in the intoxicated horses, and the absence of neutrophilic casts in the control horses (versus 4/21 (19%) cases of oleander poisoning that presented this change), which may suggest more severe tubular injury in cases of oleander poisoning. Nevertheless, these microscopic tubular changes are generally considered to be non-specific [14]. Overall, the similarities in the distribution and severity of the renal lesions between the oleander poisoning cases and the controls suggest that the changes we noted are related to cardiac failure with hypoperfusion of the renal cortex rather than a direct toxic effect of oleandrin on the tubular epithelium. In human medicine, it is accepted that acute tubular necrosis can be either ischemic or toxic, and both mechanisms may result in similar histological alterations [18]. Sepsis is hypothesized to be a cause of renal hypoperfusion, since it may reduce cardiac output, cause systemic vasodilation, and promote renal vasoconstriction [17]. This may explain the tubular abnormalities detected in some of the control animals, such as Controls 2, 7, and 10. Other causes of renal hypoperfusion include compromised intravascular volume caused by gastrointestinal losses due to diarrhea, which may explain some of the microscopic renal lesions in Control 8 [17]. The detection of renal histological lesions in some of the euthanized horses was somewhat unexpected. Perhaps the different disease processes that led to euthanasia also caused a degree of renal alteration, or maybe some of these mild findings can be considered background changes in equine kidneys (e.g., a degree of tubular hyaline cast formation could be related to unspecific accumulation of proteinaceous materials such as Tamm–Horsfall protein, as has been suggested in other species [14]). Obtaining completely healthy euthanized horses was not possible, and it was out of the scope of this study.

Tubulointerstitial mineralization was also slightly more frequent in cases of oleander poisoning than in the controls. This was one of the findings described by Renier et al. [10] in equine cases of oleander poisoning, but has not been described in cases of oleander poisoning in other species [8,11,12,13,16]. Nevertheless, the interpretation of this change in our equine cases is obscure, since a degree of tubulointerstitial mineralization is occasionally detected in horse kidneys with the absence of calcium/phosphorus imbalances (authors’ unpublished observations), perhaps related to the naturally high calcium concentration in equine urine, which could increase even more in the event of dehydration [19,20]. Having a control population with background renal histology could help to further clarify this aspect. In addition, changes in the glomeruli were rare in equines intoxicated with oleander and were even less common, proportionally, than in control cases. Lastly, the interstitial hemorrhages and marked congestion detected both in the oleander poisoning cases and in the controls could have a similar pathogenesis, which includes shock and reduced cardiac output [21]; in fact, marked congestion is virtually always present in equines that die with shock of any kind; thus, the utility of this change as a diagnostic marker is limited.

Despite the abovementioned arguments in favor of an ischemic-like effect as the most likely mechanism behind the renal lesions noted, a degree of direct toxicity of oleandrin or other toxic principles on the tubular epithelium of some cases cannot be totally excluded. Ouabain and digitalis both appear to be able to directly affect renal tissue, but these effects vary at different concentrations [7,22]. Alteration of Na^+^/K^+^-ATPase transporters in the basal membrane of the thick ascending limb of the loop of Henle and the distal convoluted tubule is a possible mechanism by which cardenolides and other cardiac glycosides may have direct toxic effects on the renal tubules [11,12,13,14,22]. However, to date, no studies have demonstrated a direct effect of oleandrin on the Na^+^/K^+^-ATPase transporters in the kidney. Oleandrin and its metabolites are primarily eliminated through the biliary system [2,5,6]. Ni et al. [6] demonstrated that less than 10% of oleandrin and its metabolites are excreted through the kidney in mice, and it is likely that most mammalian species excrete oleandrin similarly. As the serum oleandrin concentration increases, which likely occurs in experimental oleander intoxications, more oleandrin would be passing through the kidney for excretion and this could explain the greater severity of renal lesions observed in experimental intoxications in other mammals [11,12,13]. In beagle dogs intoxicated with digoxin, a cardenolide found in *Digitalis* spp., higher serum concentrations of this toxicant were related to an increase in the severity of the clinical signs, including elevations in renal biochemical markers and the increased severity of histological lesions in the kidney [23]. Oleandrin and other cardenolides might act similarly. Large doses of oleandrin lead to more acute and severe clinical signs and result in death earlier, which is similar to the observations made by Teske et al. [23] for digoxin. Lastly, the absence of consistently severe microscopic kidney changes in horses intoxicated with oleander does not necessarily preclude the occurrence of acute renal failure, since histological evidence of injury may be subtle even with striking functional impairment [18].

The limitations of this study are primarily associated with the low number of intoxicated and control animals included, and the spontaneous nature of the diagnostic cases selected retrospectively. The latter includes differences in the sampling, clinical course, and postmortem intervals, and the inability to quantify the dose of oleander each animal was exposed to or the dosing interval (i.e., if multiple exposures occurred or not), and the inability to quantify the peak serum oleandrin concentration. Further research to determine if oleandrin and other cardenolides (besides ouabain and digoxin) have direct effects on the Na^+^/K^+^-ATPase transporters in the kidneys, perhaps with an experimental model in vitro, would be beneficial for fully understanding the toxicokinetics of these compounds.

## 5. Conclusions

In cases of accidental oleander poisoning, renal gross and microscopic changes can occur in horses, as has been noted in sheep, goats, cattle, and New World camelids. The detected renal microscopic changes in accidental exposures seem to be milder and more variable than in animals of other species that were experimentally intoxicated. The microscopic renal lesions in horses do not appear to differ significantly from those observed in other diseases and are therefore unreliable postmortem diagnostic markers for oleander poisoning. Cardiac lesions continue to be the main diagnostic marker of oleander poisoning in horses.

## Figures and Tables

**Table 1:** Signalment, cause of death, and clinical history of horses with oleander poisoning and controls.

Case	Sex	Age	Breed	Cause of Death	Clinical History
1	F	7 y	American miniature horse	BE due to oleander poisoning	2 days of lethargy, inappetence, and toxemia
2	F	4 y	Pinto	Oleander poisoning	1 week of depression and inappetence
3	M	U	Morgan horse	Oleander poisoning	3 days of colic-like signs
4	M	7 y	Quarter horse	Oleander poisoning	2 days of diarrhea
5	U	10 m	Fell pony	Oleander poisoning	3 days of inappetence and diarrhea
6	M	9 m	American miniature horse	Oleander poisoning	Sudden death
7	MC	18 y	Mixed breed	Oleander poisoning	1 day of lethargy, inappetence, and endotoxemia
8	MC	16 y	Belgian horse	Oleander poisoning	2 to 3 days of colic
9	MC	8 y	U	Oleander poisoning	Found dead after feeding
10	M	2.5 y	American miniature horse	Oleander poisoning	Sudden death
11	F	Adult	Quarter horse	Oleander poisoning	1 day of colic, fever, and increased HR
12	MC	5 y	Paint horse	Oleander poisoning	1 day of inappetence and increased RR
13	MC	15 y	Hanoverian	Oleander poisoning	1 day of inappetence, lethargy, soft stools, and fever
14	F	1 y	U	Oleander poisoning	Fever, lethargy, and high and irregular HR; duration not recorded
15	MC	17 y	Quarter horse	BE due to oleander poisoning	3 days of lethargy and increased HR
16	MC	18 y	Arabian	BE due to oleander poisoning	2 days of progressive, non-responsive toxemia and dehydration
17	M	15 y	Pony	Oleander poisoning	Colic-like signs; duration not recorded
18	F	Adult	American miniature horse	BE due to oleander poisoning	2 days of inappetence, colic, and distended abdomen
19	F	3 m	Pony	Oleander poisoning	Less than 1 day of inappetence, depression, and fever
20	F	8 y	Quarter horse	Oleander poisoning	3 days of inappetence, colic, and collapse
21	MC	13 y	Paint horse	Oleander poisoning	1 day of dehydration, ileus, and increased HR
Control 1	MC	10 y	Warmblood	BE due to melanoma & CVSM	1.5 year of a mass on the neck with discomfort
Control 2	M	7 y	Thoroughbred	BE due to laminitis and synovitis	2 weeks of lameness
Control 3	F	12 y	Warmblood	BE due to CVSM	Progressive ataxia; duration not recorded
Control 4	MC	8 y	Pony	BE due to sand colic with impaction	1 h of colic
Control 5	MC	35 y	Pony	BE due to CVSM	2 weeks of progressive ataxia
Control 6	MC	5 y	Thoroughbred	Iliac artery rupture with hypovolemia	Sudden death while working
Control 7	MC	16 y	Apaloosa	Gastric rupture with sepsis/endotoxemia	Found dead
Control 8	M	1 m	American miniature horse	*Clostridioides difficile* typhlocolitis	1 week of diarrhea and dehydration
Control 9	F	9 y	Arabian	Uterine artery rupture with hypovolemia	Uterine prolapse and death 8 h after foaling
Control 10	F	4 m	Friesian	*Staphylococcus aureus* sepsis	9 days of joint effusion and fever

U: unknown; F: female; M: male; MC: male castrated; y: year-old; m: month-old; BE: barbiturate euthanasia; CVSM: cervical vertebral stenotic myelopathy; HR: heart rate; RR: respiratory rate.

**Table 2:** Histological findings in the kidneys of horses with oleander poisoning and control horses.

Case	Tubules					Glomeruli	Interstitium		Vasculature
	HC	NeC	Eat	EN	TiM	Pr	LP	He	C
1	++	−	++	−	+	+	−	−	+
2	++	−	++	+	+++	−	−	−	++
3	+	+	+	−	−	−	−	++	+++
4	++	+	+	−	++	−	−	−	+++
5	−	−	−	−	+	+	−	−	++
6	+	−	−	−	+	−	−	−	++
7	+++	−	+	−	−	−	−	−	++
8	++	+	++	−	−	−	−	−	+
9	+	−	−	+	−	−	+	+++	+++
10	+	++	+	+	+	−	+	−	+
11	+	−	−	−	−	−	+	−	+
12	+	−	−	−	−	−	−	−	+
13	+	−	−	−	+	+	−	−	+
14	+	−	−	−	++	++	−	−	+
15	+	−	−	+	+	+	−	−	++
16	+	−	+	−	−	+	+	−	++
17	+	−	+	−	+	−	−	−	+
18	−	−	−	−	−	−	+	−	+
19	−	−	−	−	+	−	−	+	+
20	+	−	−	++	+	−	−	++	+++
21	−	−	−	−	++	−	−	−	+++
Control 1	+	−	+	−	−	+	−	−	+
Control 2	++	−	+	−	−	−	−	−	+
Control 3	−	−	−	−	+	+	−	−	+++
Control 4	−	−	−	−	+	++	−	−	+
Control 5	+	−	−	−	+	+	−	−	+
Control 6	−	−	−	−	−	−	+	−	++
Control 7	++	−	−	++	+++	−	+	−	+
Control 8	+	−	+	−	−	−	−	+	+
Control 9	−	−	−	−	−	−	−	−	+
Control 10	+	−	+	+	−	−	−	+	+

HC: intratubular hyaline casts; NeC: intratubular neutrophilic casts; EAt: tubular epithelial attenuation; EN: epithelial necrosis; TiM: tubulointerstitial mineralization; Pr: proteinaceous eosinophilic material in Bowman’s space; LP: lymphoplasmacytic infiltrates; He: hemorrhages; C: Congestion; −: absence; +: mild; ++: moderate; +++: severe.

## Data Availability

The data presented in this study are available within the article.
